# Malignant Mesothelioma: Overcoming Diagnostic Hurdles

**DOI:** 10.7759/cureus.68718

**Published:** 2024-09-05

**Authors:** Priya Dharshini R, Sarah Grace Priyadarshini, Jayaganesh P

**Affiliations:** 1 Department of Pathology, Saveetha Medical College and Hospital, Saveetha Institute of Medical and Technical Sciences, Saveetha University, Chennai, IND

**Keywords:** asbestos exposure, calretinin positive, malignant pleural mesothelioma (mpm), massive pleural effusion, pleural neoplasm

## Abstract

Malignant pleural mesothelioma, an aggressive neoplasm frequently linked to asbestos exposure, is often detected at an advanced stage. This report details the case of a 58-year-old mason who presented with left-sided chest pain, and shortness of breath, accompanied by weight loss for a month. A positron emission tomography (PET) scan revealed increased uptake along the pleural surface, as well as in several mediastinal lymph nodes and the left supraclavicular lymph node. Thoracoscopy revealed the presence of multiple nodules on the costal pleura. Despite repeated negative results from pleural effusion cytology, cell block analysis, and pleural biopsies, the diagnosis of malignant mesothelioma (MM) was ultimately established through an ultrasound-guided (USG) biopsy of the left supraclavicular lymph node, with immunohistochemical confirmation using calretinin.

## Introduction

Malignant mesothelioma (MM) is an extremely aggressive malignancy with a high fatality rate [1]. It is relatively rare, with an incidence rate ranging from seven to 40 cases per million, leading to approximately 40,000 deaths globally each year [2]. Both underdiagnosis and underreporting of deaths among MM patients are widespread issues [3]. A considerable number of cases are presumed to go unreported, especially in developing regions [4].

MM originates from mesothelial cells lining the pleura and, less commonly, other serosal membranes such as the peritoneum, pericardium, and tunica vaginalis. Pleural involvement in mesothelioma accounts for up to 70% of cases, while peritoneal mesothelioma represents 30%, and pericardial mesothelioma constitutes 1-2% [5]. It can present either as a localized tumor or as a widespread involvement of the pleura or pericardium, referred to as localized or diffuse mesothelioma, respectively.

Approximately 80% of individuals with malignant pleural mesothelioma have had either direct or secondary exposure to asbestos fibers [4]. The disease generally manifests after a prolonged period, with an interval of 20-40 years following the initial exposure [4,6]. Consequently, MM is most often observed in older individuals, typically in their seventh or eighth decade, with a higher incidence in men compared to women [7].

MM can present with nonspecific symptoms such as breathlessness caused by pleural effusion, chest pain, or a dry cough. Over 80% of individuals with a history of asbestos exposure show visible pleural plaques on chest radiographs. Thoracoscopic biopsy remains the cornerstone of diagnosis, given the highly variable sensitivity of pleural fluid analysis [4].

Due to its aggressive nature, MM is often diagnosed at advanced stages, leading to significantly reduced survival rates [4]. The median survival time for patients with MM is about 15 months, and fewer than 5% of patients survive beyond five years, despite significant advances in treatment [8]. Early diagnosis is crucial for improving the prognosis for patients with MM [9].

## Case presentation

**Figure 1:**
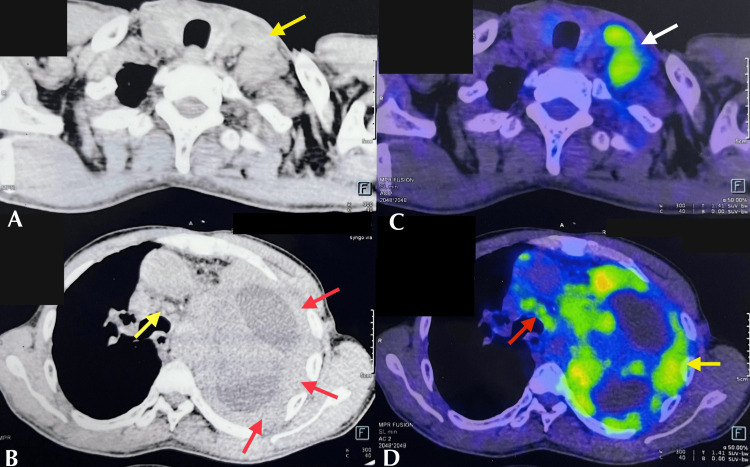
CECT and PET scan. (A) CECT showing few enlarged lymph nodes. (B) CECT showing nodular pleural thickening (red arrows) and few enlarged lymph nodes in the pre-tracheal region. (C) PET showing uptake in the left supraclavicular lymph node. (D) PET showing uptake along the pleural surface (yellow arrow) and multiple mediastinal lymph nodes (red arrow). CECT: contrast-enhanced computed tomography; PET: positron emission tomography

A 58-year-old gentleman mason by occupation presented with complaints of left-sided pleuritic chest pain with associated breathlessness and loss of weight and appetite for one month. He has been a known smoker for the past 20 years. Systemic examination and radiographs were suggestive of a left massive pleural effusion. The pleural fluid analysis did not reveal any significant findings. However, the patient's prolonged exposure to construction materials, recent history of unexplained weight loss, and decreased appetite and the presence of massive pleural effusion raised a strong suspicion of malignancy, with mesothelioma being the primary consideration. Contrast-enhanced computed tomography (CECT) and positron emission tomography (PET) scans were performed to evaluate the potential for malignancy. CECT revealed nodular pleural thickening and a few enlarged lymph nodes in the pre-tracheal and left supraclavicular regions. The PET scan showed increased uptake in the pleural surface with multiple mediastinal lymph nodes and a left supraclavicular lymph node (Figure 1).

**Figure 2:**
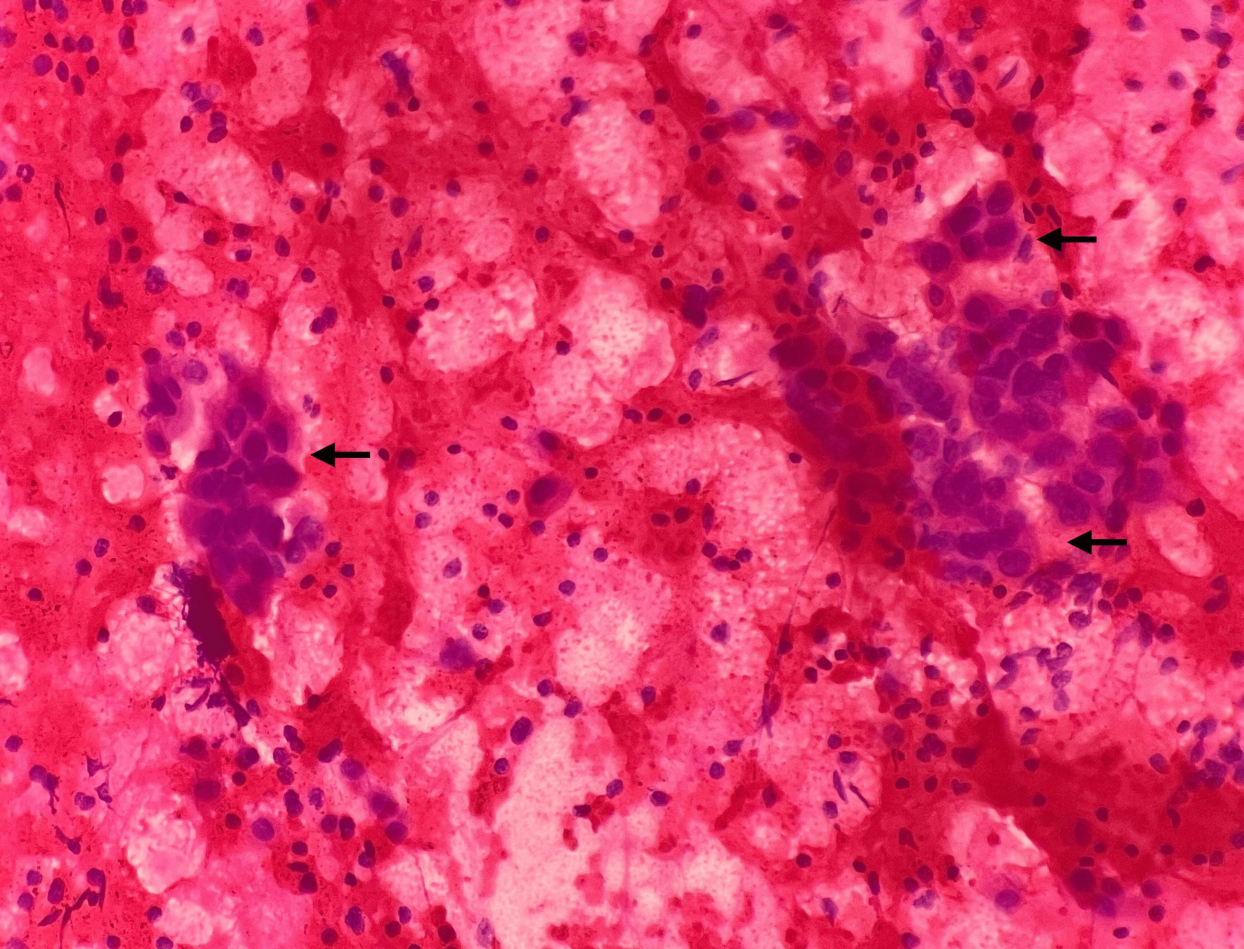
Microscopy of the USG FNAC of the left supraclavicular lymph node on high-power magnification showing features of metastatic carcinomatous deposits (H&E). USG: ultrasound-guided; H&E: hematoxylin and eosin; FNAC: fine needle aspiration cytology

The patient was taken up for thoracoscopy, and multiple nodules in a hemorrhagic background were noted along the costal pleural surface. Multiple biopsies of the nodules were sent for histopathological examination, and bronchial brush and pleural fluid samples were sent for cytology. The pleural fluid and bronchial brush samples we received showed only hemorrhagic material, negative for malignancy. The thoracoscopy-guided biopsy from the left pleura was examined at multiple deeper levels and the sections were devoid of malignant cells and repeat biopsy was advised. Repeated analyses of pleural fluid and cell block prepared were all negative for malignant cells. The ultrasound-guided (USG) fine needle aspiration cytology (FNAC) of the left supraclavicular lymph node was then performed, and the smears showed features of metastatic carcinomatous deposits (Figure 2).

**Figure 3:**
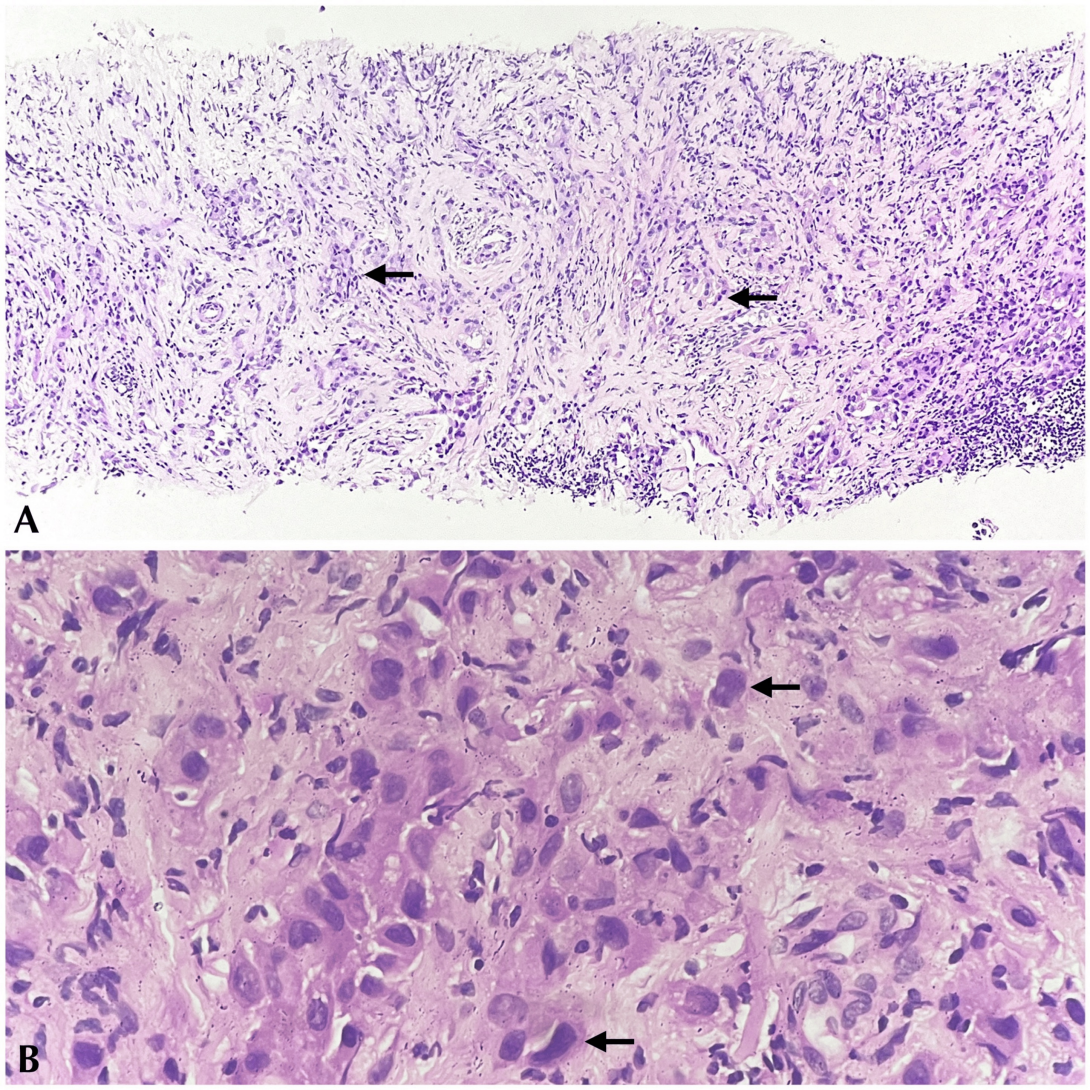
Microscopy of USG tru-cut biopsy of the left supraclavicular lymph node. (A) Low-power magnification showing tissue fragments with pleomorphic cells arranged in cords and vague glandular formation (H&E). (B) High-power magnification showing pleomorphic cells with marked nuclear atypia (H&E). USG: ultrasound-guided; H&E: hematoxylin and eosin

USG tru-cut biopsy of the left supraclavicular lymph node showed linear tissue fragments displaying cells with moderate eosinophilic cytoplasm and hyperchromatic nuclei arranged in trabeculae, sheets, and vague glandular arrangements (Figure 3).

**Figure 4:**
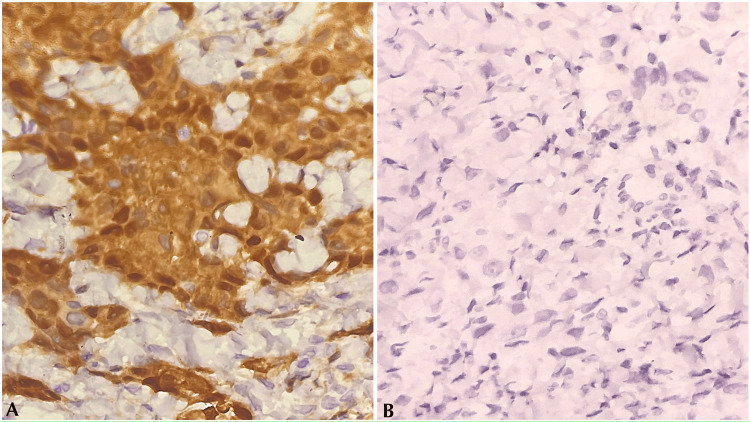
IHC of USG tru-cut biopsy of the left supraclavicular lymph node on high-power magnification. (A) Calretinin: tumor cells showing diffuse positivity. (B) TTF-1: tumor cells showing negativity. USG: ultrasound-guided; IHC: immunohistochemistry; TTF-1: thyroid transcription factor-1

A definitive diagnosis was not possible. In correlation with PET findings, an impression of MM/poorly differentiated adenocarcinoma deposits in the node was given, and immunohistochemistry (IHC) for calretinin and thyroid transcription factor-1 (TTF-1) to rule out the same was further advised. The IHC for calretinin showed diffuse positivity, whereas the IHC for TTF-1 was negative (Figure 4).

Histologically, these tumor cells were polygonal with marked nuclear atypia, arranged predominantly in trabecular pattern with focal adenomatoid and solid areas (less than 50%) exhibiting six mitoses per 10 high-power field (hpf) and no necrosis. A final diagnosis of high-grade epithelioid mesothelioma with trabecular and adenomatoid patterns was reached after correlating histological features with IHC findings. However, the observed features may not entirely represent the primary tumor characteristics, as the biopsy was obtained from a metastatic site. Nonetheless, it was included as it was the only histologic diagnosis available for our patient.

The patient was clinically diagnosed with left metastatic MM and was scheduled for palliative chemotherapy. He has completed his second cycle of chemotherapy with pemetrexed and carboplatin and is currently under follow-up with the medical oncologist.

## Discussion

The pathogenesis of MM is complex and multifactorial [10]. The majority is associated with asbestos exposure. Although MM was first described in 1931 [11], it was only formally recognized as a distinct malignancy in the late 1950s and early 1960s following a noted increase in its incidence. This surge can be attributed to the widespread industrial use of asbestos beginning in the 1920s and the long latency period associated with the disease's development [4,10]. The tumorigenesis of MM linked to asbestos exposure primarily occurs through DNA damage caused by free radicals released from the asbestos fibres [10,12]. Similarly, our patient too likely had asbestos exposure as a primary risk factor due to his extensive career in the construction sector. In addition to the environmental factors, the host's genetic landscape also plays a critical role in the development of MM. Notably, the most frequently encountered genomic alteration in MM involves a mutation in the BRCA1-associated protein 1 (BAP1) gene. This tumor suppressor gene functions as a nuclear deubiquitinating agent, primarily regulating chromatin remodeling to inhibit cell proliferation and induce apoptosis [13]. Other mutations include those in the neurofibromatosis type 2 (NF2) gene and the cyclin-dependent kinase inhibitor 2A (CDKN2A) gene [2,14].

MM initially develops in the parietal pleura, spreads locally to the visceral pleura, and subsequently involves the chest wall, diaphragm, mediastinum, and regional lymph nodes [15]. Extrathoracic lymph node involvement in MM is relatively rare [15,16]. The clinical presentation of MM varies depending on the disease's extent, ranging from symptoms such as breathlessness, chest pain, and constitutional symptoms to potentially life-threatening conditions like superior vena cava syndrome or pericardial tamponade [12,17]. In the early stages, breathlessness is usually due to pleural effusion, which is seen in 70% of individuals at presentation [12]. Thus, pleural effusion cytology is integral in diagnosing all malignant effusion cases. The sensitivity of pleural effusion cytology in detecting malignancy is 58.2%. However, the cytological yield is often low in cases of malignant pleural mesothelioma, with a sensitivity of only 28.9% [12,18]. Similarly, in our case, repeated analysis of pleural samples was negative for malignancy. Since the diagnostic yield in MM is variable, pleural biopsy though invasive is considered a gold standard investigation in MM [9].

Diagnosis of MM in pleural biopsies can be challenging. According to the 5th Edition of the WHO Classification of Tumours, Thoracic Tumours, MM has three subtypes: epithelioid, sarcomatous, and biphasic mesothelioma. Epithelioid mesothelioma is the most common subtype constituting 60-80%, characterized by a lower level of aggressiveness, a slower rate of spread, and a slightly better prognosis than the other two subtypes [2]. The epithelioid subtype displays a diverse array of cytological characteristics and histological patterns, each with distinct prognostic implications. Solid and micropapillary patterns are associated with a poor prognosis, whereas tubulopapillary, trabecular, and adenomatoid arrangements have a more favorable outcome. With respect to cytological features, a favorable prognosis is more likely when cells display low nuclear grade features and lymphohistiocytoid characteristics, while pleomorphism, high nuclear grade, and rhabdoid morphology are associated with a worse prognosis. The sarcomatoid subtype is composed of spindle cells arranged in solid sheets. The lymphohistiocytoid feature has a better outcome than the transitional type. The biphasic subtype is characterized by a blend of epithelioid and sarcomatoid components, each constituting at least 10% of the tumor [10]. In addition to the prognostic significance of cytological and histological features, poor prognosis is also associated with low mesothelin mRNA expression, a high epithelial-mesenchymal transition score, a higher score of T helper 2 cell signature, and enrichment for LATS2 mutations and CDKN2A homozygous deletion [19].

The prognosis for malignant pleural mesothelioma is exceptionally grim due to the absence of safe and effective treatment modalities. Moreover, as many patients present with advanced disease at diagnosis, the majority succumb to the illness within a year of confirmation. Thus, early diagnosis is crucial for improving patient outcomes. In cases where repeated pleural fluid cytology and pleural biopsy yield negative results, but accessible nodal uptake is observed on a PET scan, a biopsy of the node with IHC can be performed to establish a diagnosis. This approach allows for the earlier initiation of therapy, which may be beneficial for the patient.

## Conclusions

Diagnosing MM from a pleural biopsy is often challenging due to limited accessibility. This may necessitate more targeted or invasive procedures such as CT-guided biopsy or thoracoscopy. Despite obtaining the tissue, there is no certainty of a diagnosis, as the sample may or may not be adequate from the representative site, as our case illustrates. In these instances, a metastatic node, if present, can provide diagnostic clarity.
